# Gender specific age-related changes in bone density, muscle strength and functional performance in the elderly: a-10 year prospective population-based study

**DOI:** 10.1186/1471-2318-13-71

**Published:** 2013-07-06

**Authors:** Robin M Daly, Bjorn E Rosengren, Gayani Alwis, Henrik G Ahlborg, Ingemar Sernbo, Magnus K Karlsson

**Affiliations:** 1 Centre for Physical Activity and Nutrition Research, Deakin University, Melbourne, Australia; 2 Clinical and Molecular Osteoporosis Research Unit, Department of Clinical Sciences, Lund University, Lund, Sweden; 3 Department of Orthopaedics, Malmö University Hospital, Malmö, Sweden; 4 Department of Anatomy, Faculty of Medicine, Ruhuna University, 70, Galle, Sri Lanka

**Keywords:** Muscle, Functional performance, Normative aging, Balance, Bone, Gait

## Abstract

**Background:**

Age-related losses in bone mineral density (BMD), muscle strength, balance, and gait have been linked to an increased risk of falls, fractures and disability, but few prospective studies have compared the timing, rate and pattern of changes in each of these measures in middle-aged and older men and women. This is important so that targeted strategies can be developed to optimise specific musculoskeletal and functional performance measures in older adults. Thus, the aim of this 10-year prospective study was to: 1) characterize and compare age- and gender-specific changes in BMD, grip strength, balance and gait in adults aged 50 years and over, and 2) compare the relative rates of changes between each of these musculoskeletal and functional parameters with ageing.

**Methods:**

Men (n = 152) and women (n = 206) aged 50, 60, 70 and 80 years recruited for a population-based study had forearm BMD, grip strength, balance and gait velocity re-assessed after 10-years.

**Results:**

The annual loss in BMD was 0.5-0.7% greater in women compared to men aged 60 years and older (p < 0.05- < 0.001), but there were no gender differences in the rate of loss in grip strength, balance or gait. From the age of 50 years there was a consistent pattern of loss in grip strength, while the greatest deterioration in balance and gait occurred from 60 and 70 years onwards, respectively. Comparison of the changes between the different measures revealed that the annual loss in grip strength in men and women aged <70 years was 1-3% greater than the decline in BMD, balance and gait velocity.

**Conclusion:**

There were no gender differences in the timing (age) and rate (magnitude) of decline in grip strength, balance or gait in Swedish adults aged 50 years and older, but forearm BMD decreased at a greater rate in women than in men. Furthermore, there was heterogeneity in the rate of loss between the different musculoskeletal and function parameters, especially prior to the age of 70 years, with grip strength deteriorating at a greater rate than BMD, balance and gait.

## Background

Previous research has shown that age-related losses in bone mineral density (BMD), muscle strength, balance, and gait are associated with an increased risk of falls and fractures, disability, and mortality [1-4]. However, questions still remain with regard to the timing, rate and pattern of age-related changes in different measures of musculoskeletal health and function in middle-aged and older adults. While many previous cross-sectional and longitudinal studies have reported that the course of decline for measures of muscle strength, gait and balance accelerates with advancing age [3,5-11], it is evident that there is considerable heterogeneity in the timing, rate and extent of the losses among these different musculoskeletal health and functional parameters. For instance, there are reports that muscle strength may begin to decline from the age of 30 [3,12] or plateau until the fourth or fifth decade before declining thereafter [10,13]. Similarly, measures of balance, gait and mobility have been reported to deteriorate anywhere from the age of 40 to 60 years or beyond [5-7,11,14]. There are also reports that the age-related changes in these parameters are gender specific [3,7,15], although these findings are not consistent [11,13]. To our knowledge, there are no longitudinal studies which have simultaneously characterized and compared age- and gender- specific changes in BMD, muscle strength, balance and gait for 10-years of follow-up in middle-aged adults and the elderly. Determining the extent of the age-related changes in BMD, muscle strength, balance and gait speed, and whether there are gender differences and heterogeneity in the rate of decline amongst the different parameters, is important for targeted strategies to optimise musculoskeletal health and functional performance in older adults. Therefore, the aim of this prospective study was to: 1) evaluate and compare the age- and gender- specific changes in BMD, grip strength, balance, and gait speed in a population-based cohort of Swedish men and women aged 50 year and older over a 10-year period, and 2) compare the relative rates of changes between each of these musculoskeletal and functional parameters with ageing.

## Methods

### Study population

In 1988–89 a random sample of men and women born in 1908, 1918, 1928, and 1938 identified from the National Population Records of the city of Malmö and the municipality of Sjöbo, Sweden were invited to participate in this prospective, population-based study. Further details on the recruitment procedures and participant characteristics have been previously described [16]. Ten years following the initial testing (1998–99), participants were invited to a follow-up measurement. Of the 437 women and 402 men tested at baseline, 101 women and 156 men had died during follow-up according to the register of the National Swedish Board of Health and Welfare, and a further eight men and 19 women had relocated. As expected, the number of deaths increased across the age-groups over the 10-year follow-up, increasing from 2.9% and 3.8% in men and women aged 50 years at baseline to 41.7% in men and 52.9% in women initially aged 80+ years. Of the remaining 317 women and 238 men eligible for the second measurement, 206 women (65%) and 152 men (64%) agreed to return for follow-up testing. The remaining participants were either not interested, unable to be located or could not attend due to illness. The mean (±SD) follow-up period was 9.6 ± 0.3 years (range 9.0-10.8 years). This study was conducted in agreement with the Helsinki declaration with approval from the ethics committee of Lund University (LU-208-98). Written informed consent was obtained from all participants.

### Bone mineral density, grip strength, balance and gait velocity

Detailed information about the assessment of forearm BMD, grip strength, balance and gait velocity have been reported previously [16]. Briefly, BMD (mg/cm^2^) of the dominant forearm was assessed at 6 cm proximal to the ulnar styloid process by single-photon absorptiometry (SPA). Grip strength (kp/cm^2^) of the dominant hand was assessed with a medium (women) or large (men) size vigorometer (Martin Vigorometer, Germany), with the average result from three attempts (from a seated position) recorded. Balance was assessed using the standard Romberg test: 1) feet together, eyes closed (maximum 60-seconds); 2) standing on right and left leg with eyes open; and 3) standing on right and left leg with eyes closed. The time until balance was lost was recorded or a maximum of 30-seconds for each single leg stance test. Three trials were performed and the best result was recorded and the scores for the five tests were summed. Gait velocity (m/s) was tested at a distance of 30-meters with one turn in a corridor, and calculated as the distance divided by the total time to complete the test.

### Medical history, smoking status, alcohol intake and physical activity

As reported previously [16], medical history (presence of chronic diseases and use of certain medications), smoking habits (non-smokers, former or current smokers) and alcohol consumption (grams per week) were evaluated by the same questionnaire at baseline and follow-up that was developed by researchers at Lund University (Additional file 1). Physical disability was defined as having difficulty performing common activities of daily living. Disability was assessed by questionnaire by asking participants whether they: 1) required outside assistance to perform daily activities (eg. shopping, dishwashing, cleaning), and 2) could not manage activities such as shopping, dressing, making their bed or going to the toilet. If they answered “yes” to any of these questions, they were classified as physically disabled. In women, use of oral contraceptives and estrogen therapy (never or current/former) and menstrual history, including age at menarche and menopause, and history of oophorectomy, were also determined by questionnaire. Menopause was defined as occurring one year after the last menstrual period or at the time of ovariectomy. Physical activity was determined by an interview-administered questionnaire at baseline and follow-up that consisted of two components: 1) occupational activity and 2) sport/leisure time physical activity. As previously reported [16], a total activity score was calculated from both these components and participants were categorized as inactive or sedentary if they only engaged to light-moderate work and/or a sedentary or light activity lifestyle, or active if they engaged in heavy work and/or moderate or regular sports/leisure-time activity.

### Statistical analysis

All data were analysed using SPSS version 20 for Windows (SPSS, Inc, Chicago, IL, USA). Paired t-tests were used to examine gender specific within-group changes for continuous data. The McNemar test for paired proportions was used to compare the changes for categorical variables. Changes for all dependent variables were annualized and reported as a percentage, unless stated otherwise. Two-way analysis of variance (ANOVA) and covariance (ANCOVA) were used to assess interactions with the three age-change categories (50 to 60, 60 to 70 and 70+ years) and gender as fixed factors. All analyses were performed with adjustment for potential confounders, including height, rural or urban living, change in self-reported disability (unchanged or deteriorated), change in disease/medication use (unchanged or increased), change in habitual physical activity status (remained inactive, increased activity, decreased activity, remained active), menopause status (women), smoking history, and baseline values for the respective measurements. Excluding women who were current/former users of hormone therapy did not alter the results (data not shown). Gender differences for the mean changes within each age-change category were tested using ANCOVA, with adjustment for the above covariates. When overall differences were significant, the Bonferroni least significant difference (LSD) post hoc test was utilized. Differences between the rates of change in BMD, grip strength, balance and gait velocity within each gender and age-change category were assessed by paired t-tests. For this analysis, significance was set at p < 0.008 after adjustment for Bonferroni correction for multiple comparisons.

## Results

Table 1 outlines the gender-specific characteristics of the participants by the three age-change categories. Briefly, 81% of the women were postmenopausal at baseline which increased to 100% at follow-up; the proportion of women that reported using estrogen therapy also increased significantly over time in the 50 to 60 and 60 to 70 year age-categories. History of chronic disease(s)/medication use was similar in men and women at baseline and increased similarly in both genders during the 10-year follow-up. Self-reported disability was also similar between men and women at baseline, and did not increase significantly during follow-up, except in men in the 70+ age-category. A higher proportion of men at baseline were classified as former/current smokers and reported consuming at least one alcohol unit per week (both, p < 0.001). During follow-up however, there were no changes in the proportion of men or women that consumed alcohol or were classified as never, current or former smokers, with the exception of a decrease in the proportion of current smokers in men and women in the 60 to 70 and 50 to 60 year age-categories, respectively. A higher proportion of men were habitually active at baseline, but the proportion of men and women classified as active across the age-categories remained unchanged after 10-years, except for a decrease in women aged in the 60 to 70 year category. Participants that did not participate in the follow-up were significantly older, had a greater history of disease/medication use, were more likely to self-report disability, and had lower BMD and poorer function and health than those who returned (see Additional file 2: Table S1).

**Table 1:** Baseline and follow-up characteristics of the male and female study participants by birth year at baseline

	**Born 1948**		**Born 1938**		**Born 1918 to 28**	
	**Baseline**	**Follow-up**	**Baseline**	**Follow-up**	**Baseline**	**Follow-up**
**Men, n**	46		51		55	
Age (years)	50.8 ± 0.3	60.2 ± 0.4	60.6 ± 0.4	70.3 ± 0.4	72.1 ± 3.7	81.8 ± 3.7
Height (cm)	177.8 ± 6.1	177.6 ± 6.1	173.5 ± 5.6	173.6 ± 5.8	173.0 ± 5.8	172.8 ± 5.9
Weight (kg)	79.7 ± 11.8	81.1 ± 13.4	78.8 ± 10.8	79.7 ± 10.7	77.3 ± 10.6	75.1 ± 11.7**
Smoking, former/current (%)	20/24	28/15	29/35	48/22*	38/18	49/11
Alcohol, g/week	74 ± 56	81 ± 49	72 ± 54	78 ± 68	73 ± 57	76 ± 61
History of disease/medication use (%)	10.9	21.7	17.6	37.3**	29.1	54.5***
Self-reported disability (%)	2.2	2.2	7.8	5.9	5.5	18.2*
Habitual physical activity (% inactive)	50.0	65.2	47.1	51.0	50.9	67.3
Forearm BMD (mg/cm^2^)	671 ± 69	619 ± 95***	645 ± 82	595 ± 84***	609 ± 96	547 ± 101***
Grip strength (kp/cm^2^)	1.28 ± 0.21	0.90 ± 0.20***	1.19 ± 0.27	0.86 ± 0.22***	0.94 ± 0.26	0.68 ± 0.23***
Balance (seconds)	149 ± 19	143 ± 27	134 ± 22	122 ± 29**	126 ± 26	95 ± 27***
Gait velocity (m/s)	2.03 ± 0.33	2.05 ± 0.30	1.78 ± 0.30	1.69 ± 0.33	1.71 ± 0.29	1.49 ± 0.38***
**Women, n**	70		70		66	
Age (years)	51.0 ± 0.5	60.3 ± 0.4	60.7 ± 0.4	70.3 ± 0.4	72.1 ± 3.6	81.9 ± 3.6
Height (cm)	163.8 ± 5.8	163.8 ± 5.6	161.8 ± 6.7	162.2 ± 6.6	161.3 ± 5.0	161.1 ± 5.0
Weight (kg)	66.8 ± 10.3	69.8 ± 11.1***	70.1 ± 13.3	71.8 ± 15.5*	70.6 ± 13.6	67.6 ± 12.4***
Age at menarche (years)	13.6 ± 1.4	-	14.2 ± 1.5	-	14.1 ± 1.4	-
Menopause, n (%)	42.9	100	100	100	100	100
Age at menopause (years)	46.3 ± 3.6	52.6 ± 1.9	50.3 ± 3.5	-	48.5 ± 4.7	-
Years postmenopausal	4.7 ± 3.4	10.6 ± 4.0	10.4 ± 3.5	20.0 ± 3.5	23.6 ± 6.1	33.4 ± 6.1
Hormone therapy: former/current (%)	20.3	47.8***	11.4	24.3*	7.7	10.9
Smoking, former/current (%)	13/36	23/27*	14/16	19/16	11/5	11/5
Alcohol, g/week	43 ± 63	49 ± 48	44 ± 28	38 ± 18	57 ± 40	52 ± 44
History of disease/medication use (%)	17.1	34.3***	30.0	45.7**	43.1	66.7***
Self-reported disability (%)	8.6	7.1	4.3	4.3	15.2	25.8
Habitual physical activity (% inactive)	85.7	78.6	82.9	68.6*	84.8	83.3
Forearm BMD (mg/cm^2^)	527 ± 55	492 ± 77***	477 ± 61	409 ± 66***	418 ± 81	355 ± 79***
Grip strength (kp/cm^2^)	0.88 ± 0.23	0.67 ± 0.17***	0.79 ± 0.20	0.59 ± 0.15***	0.62 ± 0.12	0.47 ± 0.13***
Balance (seconds)	140 ± 20	136 ± 26	127 ± 18	107 ± 22***	113 ± 26	88 ± 28***
Gait velocity (m/s)	1.65 ± 0.32	1.64 ± 0.37	1.56 ± 0.27	1.55 ± 0.37	1.43 ± 0.24	1.18 ± 0.38***

Data are presented as means ± SD or as proportions (%); *p < 0.05, **p < 0.01, ***p < 0.001 versus baseline.

**Figure 1:**
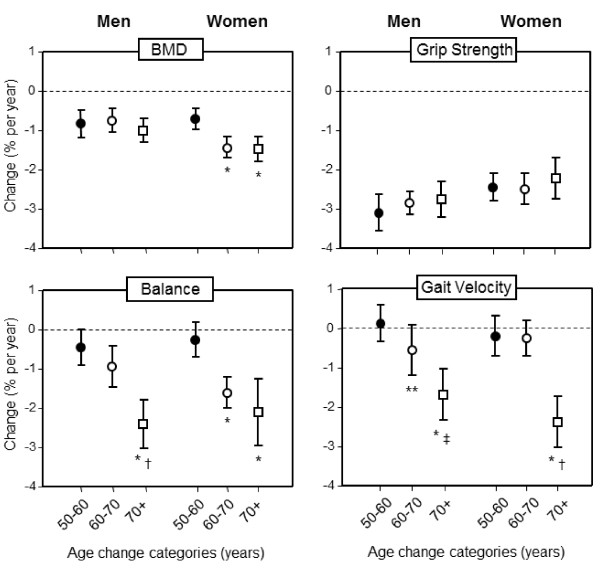
**Comparison of the mean (95% confidence interval) annual unadjusted percentage rates of change in forearm BMD, grip strength, balance and gait velocity between men and women divided into three different age-change categories.** A significant age-by-gender interaction was detected for BMD (p < 0.01). All p-values represent differences after adjusting for covariates: *p < 0.001, 70+ and 60–70 versus 50–60 years, †p < 0.001, 70+ versus 60–70 years, ‡p < 0.05, 70+ versus 50–60 years, **p < 0.05, 60–70 versus 50–60 years.

For forearm BMD, there was a significant gender by 10-year age-change interaction (p < 0.01) (Table 2 and Figure 1). Women aged 60 to 70 years and those above 70 years experienced greater losses in BMD compared to women aged 50 to 60 years. In contrast, the rate of bone loss was similar for men across the three age-change categories. For grip strength, the average loss per year was similar in each of the age-change categories for both men and women. For balance, the deterioration over time in both genders varied by age-change category, but there were no significant gender-by-age group interactions. In both men and women, there was a significant deterioration in balance over the 10-year period within each age-change category, except for women in the 50 to 60 year age category. In men, the annual deterioration was significantly greater in the 70+ compared to both the 50 to 60 and 60 to 70 year age-change categories (both p < 0.001). In contrast, for women the deterioration in balance in both the 70+ and 60 to 70 year age-change categories were greater than those observed in the 50 to 60 year age-change group (both p < 0.001). The annual percentage change for gait velocity in both genders also varied by age-change categories, but there was no significant gender-by-age group interaction. In both men and women in the 50 to 60 and 60 to 70 year age-change categories, gait velocity was similar at baseline and follow-up, but deteriorated significantly over time in the 70+ groups for both genders.

**Table 2:** Mean unadjusted annual percentage changes (95% CI) by gender and age-change categories from baseline to follow-up for bone mineral density (BMD), grip strength, balance and gait velocity

		**Age change categories from baseline to follow-up**						
**Measurements**		***N***	**50 to 60 years**	***n***	**60 to 70 years**	***n***	**70+years**	***Interaction***^***2***^
BMD (mg/cm^2^)	Men	*44*	−0.83 (−1.18, -0.46)	*48*	−0.75 (−1.05, -0.45)	*47*	−1.00 (−1.31, -0.69)	<*0*.*01*
	Women	*68*	−0.71 (−0.98, -0.43)	*68*	−1.44 (−1.71, -1.18) *	*63*	−1.48 (−1.80, -1.17) *	
	*P*-*value*^*1*^		*0*.*67*		<*0*.*001*		<*0*.*05*	
Grip strength (kp/cm^2^)	Men	*41*	−3.09 (−3.55, -2.64)	*49*	−2.84 (−3.13, -2.54)	*52*	−2.75 (−3.21, -2.28)	*0*.*92*
	Women	*67*	−2.44 (−2.78, -2.09)	*67*	−2.49 (−2.88, -2.09)	*59*	−2.22 (−2.74, -1.70)	
	*P*-*value*^*1*^		*0*.*06*		*0*.*06*		*0*.*38*	
Balance (sec)	Men	*43*	−0.46 (−0.91, 0.00)	*42*	−0.95 (−1.48, -0.42) *	*37*	−2.41 (−3.02, -1.79) *,†	*0*.*30*
	Women	*63*	−0.27 (−0.72, 0.18)	*65*	−1.61 (−2.01, -1.22) *	*36*	−2.11 (−2.95, -1.27) *	
	*P*-*value*^*1*^		*0*.*42*		*0*.*12*		*0*.*75*	
Gait velocity (m/sec)	Men	*44*	0.13 (−0.34, 0.60)	*49*	−0.55 (−1.19, 0.09) **	*50*	−1.68 (−2.33, -1.04) *,‡	*0*.*21*
	Women	*67*	−0.19 (−0.70, 0.31)	*69*	−0.25 (−0.70, 0.19)	*63*	−2.38 (−3.03, -1.74) *,†	
	*P*-*value*^*1*^		*0*.*15*		*0*.*84*		*0.86*	

^1,2^ P-values for gender differences for each age change category and the gender-by-age change category interactions were assessed by ANCOVA adjusting for covariates. *p < 0.001 versus 50–60 years; †p < 0.001 versus 60–70 years; ‡p < 0.05 versus 60–70 years; **p < 0.05 versus 50–60 years.

**Figure 2:**
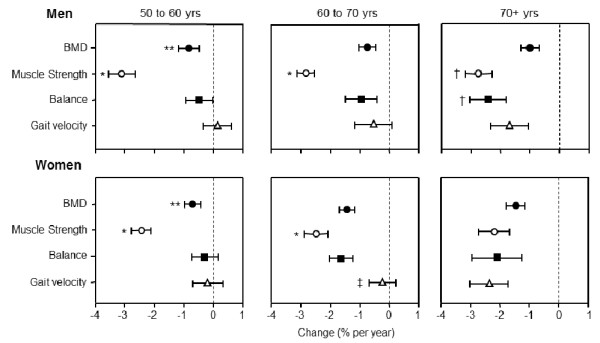
**Comparison of the mean (95% confidence interval) annual unadjusted percentage (%) rates of change between forearm BMD, grip strength, balance and gait velocity for men and women in the 50 to 60, 60 to 70 and 70+ year age-change categories.** *p < 0.001 versus the change in BMD, balance and gait velocity; **p < 0.008 versus the change in gait velocity; †p < 0.008 - <0.001 versus the change in BMD and gait velocity; ‡p < 0.001 versus the change in BMD and balance.

Comparison of the annual percentage rates of change between each of the musculoskeletal and functional parameters within each gender and age-change category revealed a greater rate of loss in grip strength relative to BMD, balance and gait for both men and women in the 50 to 60 and 60 to 70 year age change categories (all p < 0.001) (Figure 2). No other significant differences were detected within either gender or any of the age-change categories for the other variables, with the exception of the following: there was a more rapid decline in BMD relative to gait velocity for both men and women (P < 0.008) in the 50 to 60 year age-change category, and the rate of loss in BMD and balance was greater than the decline in gait velocity in women in the 60 to 70 year age-change category (both p < 0.001). For men and women in the 70+ age-change category, the annual percentage rate of loss was similar for all parameters, with the exception that the annual losses in grip strength and balance were significantly greater than the change for both BMD and gait velocity (p ranging from <0.001 to <0.008).

## Discussion

The main findings from this 10-year population-based observational study were that the mean annual percentage rate of loss in forearm BMD were 1.5 to 2.0-fold greater in women compared to men aged over 60 years, while the annual losses in grip strength, balance or gait velocity were similar in both genders. Further comparison of the changes in each of the musculoskeletal and functional parameters within each 10-year age-change category revealed that for both men and women aged <70 years, the mean annual percentage loss in grip strength was on average, 1-3% greater than the mean annual decline in BMD, balance and gait velocity. The greatest deterioration in balance and gait velocity occurred after the age of 60 and 70 years, respectively, in both men and women. Together, these findings argue for the importance of promoting targeted lifestyle and exercise interventions that focus on improving muscle strength, balance and gait in older adults, with a particular focus on optimising muscle strength from the age of 50 years and balance and gait speed from 60 years and older.

The finding that the mean annual rates of loss in forearm BMD were greater in women compared to men aged over 60 years is consistent with the results from several [17,18] but not all [19] previous prospective studies examining age-related changes in forearm BMD. For instance, in a 6-year prospective study of Norwegian women and men aged 45–84 years it was reported that the rates of bone loss at the distal forearm increased significantly across the 5-year age groups from 45–49 to 75+ years in men but not women [20]. These findings in women are perhaps not unexpected since bone loss accelerates during the menopause. However, we found that the rate of forearm bone loss was greatest in women from 60 to 70 and 70+ years; there were no significant differences in bone loss across the three age-categories in men. Although others have reported similar findings at the distal radius [18], it is possible that these mixed results may relate to the skeletal site assessed. Most previous studies have measured distal or ultradistal forearm BMD, which contains predominantly trabecular bone, whereas we assessed BMD at 6 cm proximal to the ulnar styloid process, a site consisting mostly of cortical bone. Indeed, there is evidence for a gender-specific difference in cortical and trabecular bone loss. A cross-sectional study using high-resolution quantitative computed tomography to assess distal radius cortical and trabecular bone microarchitecture in 644 Canadian adults aged 20–99 years reported that women tended to experience a greater decline in cortical thickness and cortical BMD and a greater increase in cortical porosity compared to men; trabecular bone loss was similar between men and women [21]. Since the results from this study were not presented in 5- or 10-year age categories, it is not possible to determine whether there were gender differences in cortical and trabecular bone loss after the age of 50 years. Nevertheless, these findings provide some evidence to support the greater rate of bone loss in the older women in our study.

With regard to the changes in grip strength, we found that there was a consistent rate of loss (2.2- 3.1%/year) in grip strength across the three age-change categories which did not differ by gender. However, there was a trend (P = 0.06) for a greater rate of loss in grip strength in men compared to women in the 50 to 60 and 60 to 70 year age-categories. While this is consistent with the findings from a 10-year prospective study in 120 adults aged 46–78 years which observed a greater loss in elbow extensor and flexor strength in men than women [22], the general consensus is that men and women experience similar relative losses in muscle strength [8,9,23]. In terms of the magnitude of the annual rate of loss in grip strength, many previous studies (particularly cross-sectional) have reported a mean loss ranging from 0.5% to 2.0% per year [9,10,24]. In contrast, several prospective studies conducted in both men and women have reported rates of loss ranging from 2.4% to 2.8% per annum, which is consistent with the findings from our study [8,9]. However, in most of these studies the course of the decline in strength accelerated with advancing age [3,9,10], whereas we observed a consistent pattern of loss in grip strength in both men and women from the age of 50 years. While it is difficult to explain this finding, it is possibly related to the characteristics of our cohort. The participants included in the prospective analysis were generally healthy, independent living elderly men and women and thus it is possible that they had ‘more to lose’ than a typical cohort of older adults. Indeed, those lost to follow-up were significantly older and had lower BMD and grip strength, poorer function, and poorer health than those who returned for the follow-up testing (Additional file 2: Table S1). Thus, it is possibly that our findings may be confounded by selection bias.

In terms of balance and gait, there are also inconclusive findings with regard to the age- and gender- specific patterns of loss. For instance, we found that the age (timing) and rate (magnitude) of loss in both balance and gait speed were both similar for men and women, with balance deteriorating at an earlier age than gait speed (60–70 years vs 70+ years) in both sexes. These findings are consistent with the results from a number of previous studies which have reported a curvilinear relationship between measures of gait and balance with age [5,6,15], with little evidence of a gender difference [25], and a more pronounced decline in balance/postural stability after the age of 60 [5,7] and gait speed after the age of 65 to 70 years [11,26]. Given that impaired balance and reduced gait speed have been associated with an increased risk of falls, disability and even reduced survival [1,2,4], these findings provide useful information for the optimal time to intervene with respect to targeted lifestyle and exercise strategies to optimize gait and balance in the elderly.

Previous research has shown that various measures of BMD, muscle strength, balance and gait (and their changes) are moderately correlated with each other [27], but no studies appear to have directly compared the relative rates of change (loss) between these different measures at different ages in both men and women. Our finding that the annual rate of the loss in grip strength was significantly greater than the annual decline in BMD, balance and gait velocity for both men and women in the 50 to 60 and 60 to 70 year age-change categories adds further support to current consensus guidelines recommending that middle aged and older adults partake in regular progressive resistance training to optimise muscle strength and muscle mass [28]. Similarly, the finding that the annual rate of loss in all measures tended to be similar after the age of 70 years suggests that a multi-modal exercise program targeting strength, balance and gait is needed to prevent the age-related decline in these measures in the elderly. However, when interpreting these findings it is important to note that a given percentage change in BMD may not be directly comparable (clinically) to similar percentage changes in muscle strength, balance and gait over the same period (the same applies to the changes in strength, balance and gait). As an example, a 5% loss in BMD is unlikely to be clinically comparable to a 5% decline in muscle strength over the same time. Nevertheless, we believe that these findings provide a unique insight into age-specific relative losses amongst different measures of musculoskeletal health and function in older adults.

The strengths of this study lie in its prospective population-based nature; the long-term follow-up; and the assessment of BMD, grip strength, and physical function using the same measurements and apparatus. However, there are also limitations. First, this was an observational study, and other confounders, such as dietary habits, could have influenced the inferences. However, it is worth noting that all results for all outcome variables were similar whether they were analysed unadjusted or adjusted for all potential covariates (data not shown). Second, the findings may not be applicable to other populations, such as those with a chronic disease(s) or other comorbidities, since our cohort consisted of generally healthy, independent living elderly men and women. This is confounded further by the fact that 47% of men and 38% of women from the original cohort did not return for follow-up assessment, and those lost to follow-up were significantly older, had a greater history of disease/medication use, were more likely to self-report disability, and had lower BMD and poorer function and health than those who returned (Additional file 2: Table S1). Third, it is possible that individual physical performance levels and/or health status may have changed at different times throughout the 10-year follow-up period, which may have masked the timing of the age-related changes in the different measures. Finally, the assessment of BMD was performed using SPA and limited to the forearm, and thus our findings cannot be generalized to other skeletal sites.

## Conclusions

In summary, this 10-year population-based prospective study in Swedish older adults has shown that the annual rate of loss in forearm BMD is greater in women compared to men aged 60 years and over, but that the rate and timing of loss in grip strength, balance and gait is similar in both sexes. Further comparison of the changes in each of these parameters revealed that the annual rate of loss in grip strength was greater than the annual decline in BMD, balance and gait velocity for both men and women aged <70 years. The greatest rates of deterioration in balance and gait occurred from the age of 60 and 70 years onwards, respectively. From a practical perspective, these findings provide further evidence for the importance of promoting exercise interventions to improve muscle strength from the age of 50 years and onward, with the need to develop more targeted programs designed to enhance both balance and gait speed in the elderly.

## Competing interests

The author(s) declare that they have no competing interests.

## Authors’ contributions

RMD, BER and GA analysed and interpreted the data and wrote the manuscript. IS contributed to the study concept and design, acquisition of subjects and data, manuscript review and revision. HGA and MKK were involved in drafting the manuscript and revising it critically for important intellectual content. All authors have read and approved the final version.

## Pre-publication history

The pre-publication history for this paper can be accessed here:

http://www.biomedcentral.com/1471-2318/13/71/prepub

## Supplementary Material

Additional file 1 Health, medical and lifestyle questionnaire. Click here for file

Additional file 2: Table S1 Comparison of the baseline characteristics of men and women who completed the 10-year follow-up assessment with those who were lost to follow-up. Click here for file
