# Supplementary material for: Gender specific age-related changes in bone density, muscle strength and functional performance in the elderly: a-10 year prospective population-based study
Source: BMC Geriatr. 2013 Jul 6;13:71. doi: 10.1186/1471-2318-13-71 (PMC3716823; doi:10.1186/1471-2318-13-71)
Supplement: Additional file 1 — Health, medical and lifestyle questionnaire. [file 1471-2318-13-71-S1.pdf]

APPENDIX 1.

QUESTIONNAIRE : LIFE STYLE FACTORS MALMÖ-SJÖBO

PART 1.

1. What is your education ?
1) primary school (folkskola)
2) basic school (grundskola)
3) secondary school (realskola)
4) grammar school (gymnasieskola)
5) university

2. What is your present profession, occupation ?

3. Has another profession previously been your main occupation ?
1) no
2) yes, namely.......................................

4. Which of the following describes best your physical extertion during your daily occupation at the present?
1) mainly sitting work/occupation
2) light work, but even mobile to a certain extent. Walks a bit but does not lift or carry heavy objects.
3) moderately heavy work. Much walking, lifting and climbing stairs.
4) heavy labour. Lifting heavy objects and physical exertion.

5 What description of your daily activities was most appropriate for your situation when you where 20-25 years old ?
Please use one of the alternatives from the question above.

6 What means of transportation do you mainly use in the summer ?
1) own car
2) car owned by a relative, neighbor, taxi
3) bus
4) motor cycle
5) bicycle
6) walk
7) other......................

7 What means of transportation do you mainly use in the winter ?
Please use one of the alternatives from the question above

8 Do you have community taxi service ?
1) no
2) yes

9 Do you consider that you should have community taxi service ?
1) no
2) yes

10 What is your present civil state ?
1) married/cohabitation
2) single, previously married/cohabitation
3) always been single

11 What is your present habitation ?
1) own home, flat or one family home
2) old peoples home, convalescent home or similar
3) nursing home, rehabilitation center or similar.

12 Are you living alone in your home ?
1) yes, I am living alone
2) no, I am not living alone

13 Has there been changes or discussions about changes in your habitation (on your own or others incentive) during the last six months ?
1) no
2) yes

14 If you aswered yes to the previous question, describe with your own words in what way the habitation has changed or being discussed.

15 What kind of habitation do you regard as the best for you today ?
1) the present. I am doing fine where I am now.
2) I would like to move to an other habitation
namely...(please state your preferences)

16 Where are you living presently ?
1) in a city e.g. Malmö
2) in a smaller town in the country e.g. Sjöbo
3) in a village in the country e.g. Tolånga

17 Which environment dominated the first 25 years of your life ?
1) a city environment e.g. Malmö, Helsingborg
2) a small town in the contry e.g. Simrishamn, Sjöbo
3) a country environment
4) mixed city- and country environment

18 For how long have you been living in your present environment ?
1) I have lived in the same environment as now during more than 40 years
2) I have lived in the same environment as now since 20-30 years
3) I have lived in the same environment as now since 10-20 years
4) I have lived in the same environment as now during less than 10 years

19 How much help do you presently need for managing daily activities e.g. shopping, dish-washing, cleaning, personal hygiene. ?
1) I manage by myself
2) need outside help less than four hours per week
3) need outside help on average 5-8 hours per week
4) need outside help in any form daily
5) need extended help for most of the daily activities

20 Do you manage your shopping ?
1) yes, I manange normally
2) no, I must get help

21 Do you manage to go to the WC yourself ?
1) yes, I do
2) no, I need help for that

22 Do you manage dressing and undressing yourself ?
1) yes, I do
2) no, I need help for that

23 Do you manage to do your own bed ?
1) yes, I do
2) no, I do not manage that

24 Do you think that you get sufficient help in your daily activities or do you need more ?
1) I am doing fine as it is and do not need more help
2) I have difficulties and need more help e.g home assistance

25 Do you need walking aids in the summer ?
1) no, not at all
2) yes, but only a cane outdoors
3) yes, a cane in- and outdoors
4) yes, I need two canes crutches or other such support
5) I cannot walk without a living support
6) I am bedridden/ confined to a wheelchair

26 Do you need walking aids in the winter ?
Please use one of the alternatives from the question above

27 On average, how often do you walk outdoors in the summer ?
1) daily
2) two to three times a week
3) once a week
4) seldom or never

28 On average, how often do you walk outdoors during the winter ?
Please use one of the alternatives from the question above

29 Do you usually spend your daytime out- or indoors ?
1) always indoors
2) mostly indoors
3) both

30 Did you in your past - 20 years ago- spend more time outdoors than you do today ?
1) no, no great difference
2) yes, I spent more time outdoors in the past

31 How far can/ do you walk in the winter ?
1) unrestricted distance
2) can walk outdoors but only for short distances 5-10 minutes
3) walk indoors only
4) can not walk by myself
5) bedridden/confined to wheelchair

32 How far can/ do you walk in the summer ?
Please use one of the alternatives from the question above

33 How many person live in your home ?
number..................

34 Do you have children of your own living in the same community ?

35 How much milk do you drink ?
1) at least tree litres per week
2) one to three litres per week
3) less than one litre per week

36 How much milk did you drink when you were 20 ?
Please use one of the alternatives from the question above

37 Do you exclude any food from your daily intake ?
1) meat
2) milk
3) cheese
4) gluten
5) other........................

38 What is your present weight ?

....kilogrammes

39 What was your weight at 20 ?

....kilogrammes

40 Have you at any time in your life reduced your body weight by 10% or more ?
1) no, I have never been on a diet
2) yes, but only rarely
3) yes, many times

41 What is your present height ?

....cm

42 What was your hight at 20 ?

...........................cm

43 How do you estimate your present health ?
1) good, very good
2) resonably good
3) bad, could be better
4) very bad

44 In what way do you not feel well ? (If you used alternative 3 or 4 in the previous question)
1) feel fine
2) heart ,circulatory disease
3) bronchial, lung disease
4) stomach, intestinal disease
5) urinary disease
6) muscle, joint disease
7) tiredness
8) psychiatric disease- nervousness
9) other...............................

45 Have you visited a physician during the last six months ?
1) no
2) yes

46 Have you been admitted to hospital, nursing home or similar during the last six months ?
1) no
2) yes

47 Do you feel lonely ?
1) no, never
2) only rarely
3) yes, sometimes
4) yes, often

48 Have you at any time in your life been treated with bed rest for a long time ?
1) no
2) yes, for 4 weeks or more

49 Have any of your close relatives had a fracture of the hip ?
1) no
2) yes, my mother
3) yes, my father
4) yes, a brother/sister

50 Do you suffer from vertigo, dizziness, impared balance or instability ?
1) no
2) yes, often- every week
3) yes, almost every day

51 Have you been or are presently beeing treated for epilepsia, convulsions ?
1) no
2) yes

52 Do you suffer from rheumatism or similar diseases ?
1) no
2) yes, rheumatoid arthritis
3) yes, another rheumatic disease

53 Do you suffer from diabetes ?
1) no
2) yes, insulin demanding
3) yes, tablett demanding
4) yes, but can be mananged by diet

54 Have you been treated for diseases of the thyroid gland according to the alternatives below ?
1) no
2) yes, I take Levaxin presently
3) I have previously taken Levaxin tablets
4) I have been radiated or operated for hyperthyroidism and take Levaxine tablets presently
5) I have been radiated or operated for hyperthyroidism but do not take Levaxine tablets presently

55 Do you have disorders of heart or lungs ?
1) no
2) yes, angina or heart failure
3) yes, asthma or chronic bronchitis

56 Do you fall often ?
1) no
2) only occasionally every year
3) yes, once or twice a month
4) yes, almost every week

57 If you fall- what do you think is the cause ?
....

58 Have you been treated for fractures during the last 20 years ?
1) no
2) yes

59 If you have suffered a fracture, please specify type of fracture and your age at the time of fracture.

type of fracture:	age:
....	....
....	....
....	....
....	....
....	....

60 Do you suffer from Parkinson´s disease or similar disorder ?
1) no
2) yes

61 Do you have a disorder following stroke ?
1) no
2) I have suffered a minor stroke but without residual symptoms in arms or legs
3) yes, with residual symptoms in arms or legs

62 Have you, now or previously, been treated with tablets for high blood pressure ?
1) no
2) yes, previously but not now
3) yes, i take tablets for high blood pressure

63 Have you, now or previously been treated with cortison tablets ?
1) no
2) yes, previously but not now
3) yes, I take cortisone tablets presently

64 If you use or have used cortisone tablets, for how long did the treatment last ?
....months

65 Do you use medicines e.g. Novalucol or Link, for indigestion, gastritis, gastric ulcer or heartburn ?
1) no
2) yes, but only occasionally
3) yes, during repeated periods every year
4) yes, regularly

66 Do you use homeopathic drugs ?
1) no
2) yes, sometimes
3) yes, regularly

67 What type and for what purpose ?
....

68 How is your eyesight ?
1) good, can read a newspaper
2) reduced eyesight, cannot read a newspaper without problems
3) severe eyesight problems

69 Is your hearing good ?
1) yes
2) yes, but I need a hearing aid
3) no, poor hearing even with a hearing aid

70 Do you have back-pain ?
1) no
2) yes, occasionally, every month
3) yes, occasionally, every week
4) yes, almost every day

71 Do you have hip pain ?
1) no
2) yes, occasionally, every month
3) yes, occasionally, every week
4) yes, almost every day

72 Do you have knee pain ?
1) no
2) yes, occasionally, every month
3) yes, occasionally, every week
4) yes, almost every day

73 Do you have shoulder pain ?
1) no
2) yes, occasionally, every month
3) yes, occasionally, every week
4) yes, almost every day

74 Do you smoke presently or previously, cigarettes or other tobacco products ?
1) non-smoker
2) stopped smoking at least five years ago
3) smoke, but less than 10 cigarettes a day
4) smoke at least 10 cigarettes or comparable amount of other tobacco daily

75 How much hard liqueur did you drink on average per week last year ?
1) none
2) 50 ml
3)100 ml
4)150 ml
5) 250 ml
6) 370 ml
7) 500 ml
8) 750 ml or one whole bottle
9) 1100 ml
10 1500 ml or more

76 How much wine (Sherry, Port) did you drink on average per week last year ?
1) none
2) 150 ml (1-2 glasses of wine)
3) 370 ml
4) 500 ml
5) 750 ml (1 whole bottle)
6) 1100 ml
7) 1500 ml
8) 2250 ml (3 bottles of wine)
9) 3000 ml
10) more than 3000 ml per week (more than 4 bottles)

77 How much regular wine did you drink on average per week last year ?
Please use one of the alternatives above.

78 How much strong beer did you drink on average per week last year ?
1) none
2) one can a 450 ml (1.5 bottles a 330 ml)
3) three cans a 450 ml
4) five cans a 450 ml
5) seven cans a 450 ml
6) fifteen cans a 450 ml
8) at lest 25 cans a 450 ml per week (34 or more bottles a 330 ml)

79 How much beer did you drink on average per week last year ?
Please use one of the alternatives above.

80 Have you had a cancer diagnosed or being treated for cancer ?
1) no
2) yes

81 If you aswered yes, what type of cancer ?
specify...

82 What is the condition of your teeth ?
1) good
2) poor, but still there
3) dentures
4) no teeth

83 How much coffee do you drink
1) less than 4 cups a day
2) more than four cups a day
3) unknown

84 Have you been granted an early retirement ?
1) no
2) yes

85 Have you been operated on with a resection of a part of your stomach ?
1) no
2) yes

86 Have you been treated for a kidney stone ?
1) no
2) yes

The following questions are only to be answered by women

87 Have you been operated on with removal of both ovaries ?
1) no
2) yes

88 If both ovaries have been removed, how old were you then ?
....

89 How old were you when you had your first period ?
....

90 How old were you when the periods stopped ?
....

91 When you had periods, how many did you have per year on average ?
....

92 How many times have you been pregnant ?
....

93 How many children did you raise ?
....

94 Have you taken female sex homones/estrogenes
1) no
2) yes, estrogenes
3) yes, oral contraceptives
4) yes, presently estrogenes
5) yes, presently oral contraceptives

QUESTIONS ANSWERED AT THE INTERVEW:

95 Ethnic group
1) Scandinavian
2) Caucasian
3) other

96 Mental status
1) orientated in time and space. Knows name, age, address, weekday and the name of the king
2) orientated only in known surroundings. Knows name, birthyear, address but not weekday or the name of the king
3) defective orientation even in known surroundings, Knows season and name but not birthyear or address
4) completely disorientated in time and space

97 ACTIVITY LEVEL/ WORK during age 15-30
1) light work e.g office clerk, teacher, civil servant
2) medium work e.g., nursing, driver, cleaner
3) heavy e.g., farmer, construction worker, manual labour

98 ACTIVITY LEVEL/ WORK during age 30-45
Classification as in 97

99 ACTIVITY LEVEL/ WORK during age 45-65
Classification as in 97

100 ACTIVITY LEVEL/ WORK after the age of 65
Classification as in 97

101 TRANSPORTATION during ages 15-30
1) own car
2) bus or train
3) walk or bicycle

102 TRANSPORTATION during ages 30-45
Classification as in 101

103 TRANSPORTATION during ages 45-65
Classification as in 101

104 TRANSPORTATION after the age of 65
Classification as in 101

105 HOUSING during ages 15-30
1) modern flat
2) modern flat and a summer home
3) one family house
4) one family house and a summer home

106 HOUSING during ages 30-45
Classification as in 105

107 HOUSING during ages 45-65
Classification as in 105

108 HOUSING after the age of 65
Classification as in 105

109 SPARE TIME during the ages 15-30
Do you gymnastizise, run or practice any form of sport or gardening ?
1) no
2) yes- moderate exercise such as cycling, walking or lighter gardening 1-2 times per week
3) yes- regular exercise in various sports, aerobics or heavy gardening at least 3-4 hours weekly
4) yes- hard exercise and competitive sports regularly and many times weekly

110 SPARE TIME during the ages 30-45
Classification as in 109

111 SPARE TIME during the ages 45-65
Classification as in 109

112 SPARE TIME after the age of 65
Classification as in 109
