# Supplementary material for: Gender specific age-related changes in bone density, muscle strength and functional performance in the elderly: a-10 year prospective population-based study
Source: BMC Geriatr. 2013 Jul 6;13:71. doi: 10.1186/1471-2318-13-71 (PMC3716823; doi:10.1186/1471-2318-13-71)
Supplement: Additional file 2: Table S1 — Comparison of the baseline characteristics of men and women who completed the 10-year follow-up assessment with those who were lost to follow-up. [file 1471-2318-13-71-S2.docx]

Supplementary Table 1. Comparison of the baseline characteristics of men and women who completed the 10-year follow-up assessment with those who were lost to follow-up.

|  | **Men** | | **Women** | |
| --- | --- | --- | --- | --- |
|  | **Included** | **Lost to Follow-up** | **Included** | **Lost to Follow-up** |
| N | 152 | 250 | 206 | 231 |
| Age (years) | 61.8 ± 9.0 | 69.4 ± 9.9 *** | 61.0 ± 8.8 | 68.7 ± 10.6 *** |
| Height (cm) | 174.6 ± 6.2 | 173.9 ± 6.0 | 162.3 ± 6.0 | 160.6 ± 6.2 ** |
| Weight (kg) | 78.5 ± 11.1 | 78.2 ± 11.9 | 69.1 ± 12.6 | 67.6 ± 12.0 |
| Age at menarche (years) | - | - | 13.9 ± 1.4 | 14.1 ± 1.5 |
| Menopause, n (%) | - | - | 166 (81) | 211 (91) ** |
| Age at menopause (years) | - | - | 48.8 ± 4.3 | 48.5 ± 5.0 |
| Hormone therapy: former / current (%) | - | - | 13 | 5 ** |
| Smoking, former / current (%) | 56 | 66 | 32 | 28 |
| Alcohol, g/week | 73 ± 55 | 72 ± 63 | 46 ± 48 | 48 43 |
| History of disease / medication use (%) | 19.7 | 46.2 *** | 28.2 | 44.7 ** |
| Self-reported disability (%) | 5.3 | 13.6 ** | 9.2 | 24.5 *** |
| Habitual physical activity (% inactive) | 49.3 | 66.0 ** | 85.5 | 89.3 |
| Forearm BMD (mg/cm^2^) | 641 ± 87 | 600 ± 87 *** | 475 ± 80 | 434 ± 90 *** |
| Grip strength (kg/cm^2^) | 1.12 ± 0.29 | 0.94 ± 0.32 *** | 0.77 ± 0.22 | 0.62 ± 0.25 *** |
| Balance (seconds) | 137 ± 24 | 105 ± 0.39 *** | 129 ± 23 | 105 ± 34 *** |
| Gait velocity (m/s) | 1.83 ± 0.33 | 1.54 ± 0.39 *** | 1.55 ± 0.29 | 1.35 ± 0.35 *** |

Data are presented as means±SD or as proportions (%); **p<0.01, ***p<0.001 versus those included in the study with 10-year follow-up data.
